# Phytochemical analysis and biological activities of *in vitro* cultured *Nidularium procerum*, a bromeliad vulnerable to extinction

**DOI:** 10.1038/s41598-020-64026-z

**Published:** 2020-04-24

**Authors:** André Luiz Gollo, Valcineide O. A. Tanobe, Gilberto Vinícius de Melo Pereira, Oranys Marin, Sandro José Ribeiro Bonatto, Suzany Silva, Ivan Ricardo de Barros, Carlos Ricardo Soccol

**Affiliations:** 1 Department of Engineering and Biotechnology, Federal University of Paraná, CEP, 81531-980 Curitiba, Paraná Brazil; 2 Department of Chemistry. Centro Universitario de Ciencias Exactas e Ingenierías – CUCEI. C.P.44430. Guadalajara University, Guadalajara, Jalisco Mexico; 3 Instituto de Pesquisa Pelé Pequeno Príncipe and Faculdades Pequeno Príncipe, Curitiba, Paraná Brazil; 4 Postgraduate Program in Chemical Engineering, Federal University of Paraná, P.O. Box 19001, Centro Politécnico, CEP, 81531-980 Curitiba, Paraná Brazil

**Keywords:** Biotechnology, Field trials

## Abstract

This study reports the first phytochemical and biological characterization in treatment of adrenocortical carcinoma cells (H295R) of extracts from *Nidularium procerum*, an endemic bromeliad of Atlantic Forest vulnerable to extinction. Extracts of dry leaves obtained from *in vitro*-grown plants were recovered by different extraction methods, *viz*., hexanoic, ethanolic, and hot and cold aqueous. Chromatography–based metabolite profiling and chemical reaction methods revealed the presence of flavonoids, steroids, lipids, vitamins, among other antioxidant and antitumor biomolecules. Eicosanoic and tricosanoic acids, α-Tocopherol (vitamin E) and scutellarein were, for the first time, described in the *Nidularium* group. Ethanolic and aqueous extracts contained the highest phenolic content (107.3 mg of GAE.100 g^−1^) and 2,2-diphenyl-1-picryl-hydrazyl-hydrate (DPPH) radical scavenging activity, respectively. The immunomodulatory and antitumoral activities of aqueous extracts were assessed using specific tests of murine macrophages modulation (RAW 264.7) and 3-(4,5-dimethylthiazol-2-yl)-2,5-diphenyltetrazolium bromide (MTT) assay against adrenocortical carcinoma cell line, respectively. The aqueous extract improved cell adhesion and phagocytic activities and phagolysossomal formation of murine macrophages. This constitutes new data on the Bromeliaceae family, which should be better exploited to the production of new phytomedicines for pharmacological uses.

## Introduction

Bromeliaceae is a morphologically distinctive and ecologically diverse family, divided into eight subfamilies (Brochinioideae, Lindmanioideae, Tillandsioideae, Hechtioideae, Navioideae, Pitcairnioideae, Puvoideae and Bromeliodeae) based on morphological and molecular DNA data^1^. Almost the entire family is native to the American continent, with the exception of *Pitcairnia feliciana* (A.Chev), an endemic specie of West Africa^2^. Due to their wide distribution and abundance in tropical habitats, bromeliads represent a very important ecological component in many communities, with a direct impact on richness and diversity of fauna and flora^3^.

Bromeliads are also worldwide recognized for their ornamental value. In the past decades, it has become very popular as a garden plant, which increased the extraction pressures from natural populations. Brazil is the diversity center of Bromeliaceae, with 1,246 species cataloged to date, in which, 1,067 are endemic to the country^4^. Among these, six species are classified as vulnerable, three endangered and seven critically endangered, indicating threatened ecosystems according IUNC criteria^5^. *Nidularium* is a genus with high vulnerability to extinction and *Nidularium procerum* Lindm is one of the most prevalent bromeliads found in the Atlantic Rain Forest^6^. It is a polymorphic specie, with varying appearance in response to the environment, especially the coloration of the leaves and bracts involved. The populations are mainly concentrated on the coast, where they develop in isolation or in groups of 2–10 individuals^7^.

The bromeliad family has been used for centuries in Native American medicine^8^. More recent research has confirmed the beneficial effects of bromeliads supported by traditional medicine, such as improvement of digestive, diuretic and respiratory processes^9,10^. Other biological actions include relief of fever symptoms and diabetes mellitus^11^, as well as anti-inflammatory and anti-allergic proprieties, being able to inhibit the influx of pleural neutrophils and mononuclear cells in allergy-induced mice and, also, decrease the number of eosinophils by inhibiting PAF and eotaxin-induced eosinophil chemotaxis^12,13^. In addition, bromeliad extracts are reported and used as antitumoral agents. Bromelain and fastuosain — a complex natural mixture of proteolytic enzymes described in the group — was demonstrated to induce the apoptosis pathway of human epidermoid carcinoma and melanoma cells^14,15^. Phytochemical compounds present in bromeliads family were also shown to affect cell adhesion molecules involved in other pathways of carcinoma cells growth^16^.

In the current scenario of vulnerability caused by human exploitation, it is necessary to use alternative methods that allow cultivation of plant species. Thus, *in vitro* plant tissue culture represents an ecological alternative to obtain competent explants (plant parts) under controlled conditions. This cultivation system allows the obtaining of several compounds with pharmacological interest without affecting natural population levels. In addition, micropropagated plants produce secondary metabolites at an early stage of growth^17^, which can be a way to provide rapid propagation of a large number of uniform plants, without being affected by adverse natural factors, such as climate, season, diseases and slow plant growth^18^. This allows for a technology alternative for rapid production of pharmacological compounds that can be utilized for medicinal purpose.

Recent studies have revealed the pharmacological properties of *N. procerum*. Amendoeira *et al*.^12^, Amendoeira *et al*.^19^ and Vieira-de-Abreu *et al*.^13^ reported that extracts of *N. procerum* have analgesic, anti-inflammatory and antiallergic properties with nontoxic activities, making it an attractive candidate for future drug development. However, the phytochemical composition of *N. procerum* remains poorly studied. Only a study conducted by Williams (1978)^20^ reported the flavonoid composition of leaves of *Bromelia* spp. including *N. procerum*, which observed the presence of quercetin. Here, crude extracts from the leaves of *in vitro* cultivated *N. procerum* were analyzed by diverse chemical analysis, including phytochemical screening by colorimetric tests, target compounds by gas chromatography assays (GC), chlorophyll quantification, total phenolic compounds and individual phenols by high performance liquid chromatography (HPLC), and antioxidant activity. In addition, for the first time, the immunomodulatory and antitumoral activities of aqueous extract of *N. procerum* leaves were assessed using specific tests of murine macrophages modulation and MTT assay against adrenocortical carcinoma, a cancer with rare treatment.

## Results and Discussion

### Phytochemical screening

The phytochemical screening of crude extracts from the leaves of *in vitro* cultivated *N. procerum* revealed the presence of some secondary metabolites, according to the solvent used (Table 1). The Ethanolic (ET), Hot Aqueous (HA-100 °C) and Cold Aqueous (CA-25 °C) extracts showed positive results for alkaloids, with the appearance of red orange precipitated complexes on Dragendorff test. Two qualitative tests (i.e., Wagner and Mayer) were also carried out for this chemical group, confirming the positive results. Furthermore, the aqueous extracts showed defined turbidity (HA) and precipitate (CA), whereas ET showed only opalescence, indicating that aqueous extracts could also be effective for alkaloid extraction.

**Table 1:** Preliminary screaning of hexane, ethanolic and aqueous extracts of *Nidularium procerum* Lindm shoots.

Metabolites	Hexane Extract	Ethanolic Extract	Hot Aqueous Extract	Cold Aqueous Extract
Alkaloids Dragendorff	−	+	++	+++
Alkaloids Wagner	−	+	++	+++
Alkaloids Mayer	−	+	++	+++
Reducing Sugars	−	−	−	−
Quinones	−	−	−	−
Saponins	−	−	−	−
Mucilages		−	−	−
Coumarins	−	−	−	−
Steroids/Triterpenoids	++	+	−	−
Resins	−	−	−	−
Flavonoids		+	++	++
Tannins	−	++	+	+

Blank spaces mean that the test was not performed on the extract.

(+) small quantity positive response was obtained for the chemical group in the extract.

(++) medium quantity positive response was obtained for the chemical group in the extract.

(+++) positive response of greater quantity was obtained for the chemical group in the extract.

(−) negative response was obtained for that chemical group in the extract.

The Liberman-Buchard test revealed the presence of steroids/triterpenoids in Hexanoic (HE) and ET extracts. HE showed the formation of yellow color, indicating the possible presence of a methyl group on carbon 14^21^. In the ET, a green color was observed, related with a carbonyl function in carbon 3 and double bound between carbons 5 and 6 or 7 and 8 in the extracted compounds^22^.

The flavonoid group was observed in ET, HA and CA extracts, with the appearance of yellowish green and intense yellow, respectively, by Shinoda test. In addition, the ferric chloride assay confirmed the presence of tannins in these extracts. The ET showed a blueish black color, indicating the presence of pyrogallol tannins or hydrolysable tannin, which generates gallic acid or ellagic acid when hydrolyzed by acids, bases or appropriated enzymes^23^. Finally, both aqueous extracts showed intense green color, referring to the presence of pyrocatecholic tannins (condensed tannins), formed by condensation of two or more flavanols, which are not hydrolyzed by acids, bases or specific enzymes^23^.

These secondary metabolites found in the *N. procerum* extracts are important due to their biological activities. Alkaloids, flavonoids, tannins and other biomolecules are known for their antioxidant, antifungal, anticancer, antiviral, anti-inflammatory and antiophidic activities^24–26^. Moreover, previous studies reported the presence of sterols, di and triterpenes, phenolic compounds, flavonoids, lignin, saponins, coumarins and cinnamic acids derivatives in other species grown under natural conditions of the Bromeliaceae family^6,27^.

### Gas Cromatography – Mass Spectroscopy (CG-MS)

GC-MS was carried out in order to describe and quantify the compounds found in a solvent polarity gradient, chosen according to permission of method. A total of 43 phytocompounds were found (Table 2). These compounds belong to different chemical classes, including hydrocarbons, esters of fatty acids, steroids/triterpenes, aldehydes, amides, vitamins and flavones. The highest number of compounds (28) was evidenced in chloroform leaf extract (CHL), followed by methanol (ME) (11) and hexane (4). The major compounds found were hydrocarbons (32,5%), esters of fatty acids (21%) and steroids/triterpenes (9,3%). Some of these compounds, such as tetrapentacontane, tetradecane, stigmasterol, neoftadiene (7,11,15-trimethyl-3-methylidenehexadec-1-ene), n-hexadecanoic acid, oleic acid and octadecanoic acid, have already been reported from leaves extracts of other plant species^28^. Steroids (β-cytosterol and stigmasterol) and lipids (palmitic acid, oleic acid, γ-tocopherol, α-tocopherol) were described in *Bromelia Laciniosa* Mart. ex Shult. & Schult.f, *Neoglaziovia variegata* Mez and *Encholirium spectabile* Schult. & Schult^2^, while cyclolaudenol triterpene was reported in *Tillandsia fasciculata* Sw. hexanoic extract^29^.

**Table 2:** Main compounds found in different extracts of *N. procerum* Lindm by GC-MS.

Coumpounds	Chemical Class	Area	*m/z*	Retencion time
**Chloroform**				
7,11,15-trimethyl-3-methylidenehexadec-1-ene	Hydrocarbon	5329	68.0	19.813
Methyl hexadecanoate	Fatty Acid Ester	10533	74.0	21.079
Tetradecan-2-ylbenzene	Hydrocarbon	59459	105.0	22.299
Methyl (9*E*,12*E*)-octadeca-9,12-dienoate	Fatty Acid Ester	2038	67.0	23.357
1-hexadecanoyloxy-3-hydroxypropan-2-yl)hexadecanoate	Fatty Acid Ester	24428	57.0	25.871
(*Z*)-octadec-9-enamide	Amide	35867	59.0	26.662
1-iodotriacontane	Hydrocarbon	38790	57.0	27.020
(2-hydroxy-3-octadecanoyloxypropyl) octadecanoate	Fatty Acid Ester	9716	57.0	28.218
Tetracontane	Hydrocarbon	56949	57.0	29.220
(Z)-9-Octadecenoic acid 1,2,3-propanetriyl ester	Fatty Acid Ester	14734	55.0	29.954
(*E*)-octadec-9-enal	Aldheyde	14614	55.0	30.112
(1*R*,4*R*)-3,3,4-trimethyl-4-(4-methylphenyl)cyclopentan-1-ol	Hydrocarbon	3318	147.0	30.717
(*Z*)-docos-13-enamide	Amide	56502	59.0	31.153
3-[(*E*)-dodec-2-enyl]oxolane-2,5-dione	NC*	10118	67.0	31.158
2-methyl-3-(4-propan-2-ylphenyl)propanal	Aldheyde	39439	133.0	31.253
1-chloroheptacosane	Hydrocarbon	51728	57.0	31.267
2-octyl-3-pentadecyloxirane	Hydrocarbon	9077	55.0	31.270
3,4-dihexyl-7,7-dimethylcyclohepta-1,3,5-triene	Hydrocarbon	18042	119.0	31.398
12-[(2*S*,3*R*)-3-octyloxiran-2-yl]dodecanoic acid	Hydrocarbon	6008	67.0	31.655
4-methyl-2-[(2,4,6-trimethylphenyl)methylsulfanyl]-1*H*-pyrimidin-6-one	NC	67968	133.0	31.944
Tetrapentacontane	Hydrocarbon	88322	57.0	32.247
Dotriacontane	Hydrocarbon	14947	57.0	34.587
4-*O*-(2,2-dichloroethyl) 1-*O*-undecyl (*E*)-but-2-enedioate	Fatty Acid Ester	26373	69.0	34.595
α-Tocopherol-β-D-mannoside	Vitamin	10086	165.0	35.329
5,6,7-trimethoxy-2-(4-methoxyphenyl)chromen-4-one	Flavone	52630	327.0	36.140
β sitoesterol	Steroid/Triterpene	2386	135.0	38.729
Cyclolaudenol	Steroid/Triterpene	7665	207.0	41.783
Stigmasterol	Steroid/Triterpene	34250	95.0	43.329
**Methanol**				
1-(4-hydroxy-2-methylphenyl)ethanone	Acetophenone	66807	150.0	13.982
2-(hydroxymethyl)-2-nitropropane-1,3-diol	Alcohol	25556	57.00	16.115
Trehalose	Saccharide	56984	73.00	19.635
Methyl hexadecanoate	Fatty Acid Ester	13085	74.00	23.918
Methyl (9*E*,12*E*)-octadeca-9,12-dienoate	Fatty Acid Ester	3758	67.00	26.202
Methyl octadeca-9,12,15-trienoate	Fatty Acid Ester	7985	79.00	26.292
(4*R*)-2-methylpentane-2,4-diol	Alcohol	10890	59.00	5.676
1-(3-methoxyphenyl)ethanone	Acetophenone	1754	44.00	14.113
2-chloro-4-methylpentan-3-ol	Alcohol	7720	57.00	16.126
β-D-galactopyranosyl-(1 → 4)-D-glucose	Saccharide	11882	73.00	19.655
[(E)-henicos-10-en-11-yl]benzene	Hydrocarbon	6891	118.0	24.983
**Hexan**				
Tetradecan-2-ylbenzene	Hydrocarbon	12199	105.0	20.874
Tetrapentacontane	Hydrocarbon	42750	57.0	34.579
α-Tocopherol-β-D-mannoside	Vitamin	42982	165.0	35.317
Stigmasterol	Steroid/Triterpene	77970	95.0	43.328

*NC – Not Classified.

The most commonly foun sterols in plants include campesterol, sitosterol and stigmasterol^30^. Stigmasterol, in particular, has been investigated for its pharmacological potential, including cytotoxic, antioxidant, antitumoral, antimutagenic, among other herbal approaches to pathological states in principles and practice of phytotherapies^31^. Furthermore, to the best of our knowledge, it is the first time that some important compounds, already described in the literature in other plant species, were reported in the *Nidularium* group. These include α-Tocopherol (vitamin E) and the 5,6,7-trimethoxy-2-(4-methoxyphenyl)chromen-4-one flavone, also known as scuttelarein. Previously, α-Tocopherol was reported in four species of the family *B. laciniosa* (1.8% content in hexane leaves extract), *N. variegate* (1.5% content in hexane leaves extract), *E. spectabile* (0,9% content in hexane leaves extract) and *Ananas erectifolius* (31.4 mg/Kg of fiber)^2,32^. The presence of vitamin E in *N. procerum* crude extracts increase the antioxidant and nutritional importance of the specie. In addition, scutellarein, previously found in *Pitcairnia darblayana* Sallier, *Pitcairnia poortmanii* André, *Pitcairnia xanthocalyx* Mart., *Pitcairnia corallina* Linden at André, *Pitcairnia punicea* Schiedw^20^ and *Bromelia pinguin* L. bromeliads^33^, were also related as anticancer agent against fibrosarcoma cells, by induction of cells apoptosis pathway^34^.

### Lipid profile – gas cromatography (GC-MS)

The lipidic profile of *N. procerum* was investigated in the same apolar extractors used in GC-MS assay, whereas in the polar phase, it was analyzed in aqueous solvents, used in biological tests. The major identified constituents were Linolelaidic acid methyl ester (C18:2 – Omega 6), Methyl palmitate (C16:0 – Palmitic Acid), Cis-9-Oleic acid methyl ester (C18:1 – Oleic acid) and ɣ-Linolenic acid methyl ester (C18:3 – Gamma linolenic acid/Omega 6) (Table 3). Omega-6 Linolelaidic acid, an isomer of Linoleic acid found in spinach, broccoli, potatoes soya bean, cotton seed oil and sunflower oil^35^, was the major component found in the extracts. Furthermore, palmitic, oleic and ɣ-Linolenic acids are lipidic compounds commonly detected in Bromeliads^36^

**Table 3:** Percentage of fatty acid in relation to the total fatty acids present in the hexane, chloroform, hot aqueous and cold aqueous extracts of fresh *N. procerum* Lindm plants multiplied on MS after 90 days of i*n vitro* culture.

Esters obtained from fatty acids	% of Fatty Acids			
	Hexane	Chloroform	HA	CA
Methyl palmitate (C16:0) (*Palmitic Acid*)	zdc	24.08	23.25	20.78
Methyl palmitoleate (C16:1) (*Palmitoleic Acid*)	5.87	2.86	5.36	6.66
Methyl heptadecanoate (C17:0) (*Margaric Acid*)	ND	ND	4.16	ND
Methyl octadecanoate (C18:0) (*Stearic Acid*)	4.62	9.10	ND	8.20
cis-9-Oleic acid methyl ester (C18:1_cis9) (*Oleic Acid*)	15.81	10.25	34.86	24.12
Linolelaidic acid methyl ester (C18:2) (*Omega-6*)	28.65	21.95	27.72	21.19
Methyl Arachidate (C20:0) (*Eicosanoic Acid*)	8.78	8.56	0	10.53
ɣ -Linolenic acid methyl ester (C18:3 n-6) (*Omega-6*)	14.71	15.54	4.64	8.52
Methyl tricosanoate (C23:0) (*Tricosanoic Acid*)	1.79	7.66	ND	ND

Other minor constituents detected were stearic and palmitoleic acids, previously reported in *A. erctifolius* L.B.Sm.^32^*, B. pinguin* L.^36^, *B. laciniosa* Mart. ex Shult. & Schult.f*, N. variegata* Mez and *E. spectabile* Schult. & Schult. bromeliads^2^. Moreover, eicosanoic and tricosanoic acids were also described (Table 3) and, to our knowledge, it was the first time these compounds are found in bromeliads leaves extracts. Eicoisanoic acid was already described in *A. erectifolius* bromeliad (24,2 mk/Kg in Fibers)^32^ and reported for having anticancer and antiinflamatory potential^37^; while tricosanoic acid was present in hexane extract of leaves from *Ananas cosmosus* bromeliad, which demonstrated potential cytotoxic against tumoral cell lines^38^.

Fatty acids play an important role in biological functions of living organisms, contributing to the prevention and treatment of some diseases. Diets with oleic, linolenic, linoleic and linoleic conjugates have been shown to reduce plasma cholesterol levels, in addition to affecting some physiological reactions, such as immune response and inhibition of tumor growth^39^, decreased risk of coronary heart disease, and protective action against stroke, age-related cognitive decline and Alzheimer disease^40,41^. Moreover, Omega-6 fatty acids has gained attention in medicine studies for showing potential in preventing sarcopenia, modulate cancer, atherosclerosis, obesity, immune function and diabetes^40,42^. This is the first time the lipid profile of *N. procerum* was described, improving the knowledge of its chemical compounds.

### Chlorophyll quantification

Leaves of *in vitro* cultivated *N. procerum* showed a greater concentration of chlorophyll b (215.06 ± 14.8 µg.g^−1^ of fresh mass) than chlorophyll a (170.75 ± 18.5 µg.g^−1^ of fresh leaves). In comparison with *Nidularium campo–alegrense* Lem (56.4 ± 11.2 µg.g^−1^ fresh mass) and *Aechmea ornate* Baker (97.1 ± 11.2 µg.g^−1^ fresh mass) wild-type bromeliads^43^*, N. procerum* presented higher amounts of both chlorophylls. The photosynthetic mechanism of plants grown during *in vitro* culture is not completely active and the leaves have a reduced capacity to synthesize organic compounds^44^. Then, plants can compensate this failure with greater amounts of photosynthetic pigments, as they tend to increase its concentration, with reduced light intensity. Furthermore, bromeliads usually grow under the canopy and the leaves of shade plants often have higher content of chlorophylls than sun species^45^.

Despite the low ratio between chlorophylls a/b (0.79 ± 0.1 µg.g^−1^), when compared to *N. campo – alegrense* Lem (3.07 µg.g^−1^) and *A. ornate* Baker (2.94 µg.g^−1^) grown in normal conditions^43^, the higher amount of pigments can be related to the improvement of biological activity of the extracts. Chlorophyll compounds have been described as potential antioxidants with effective activity against lipidic peroxidation, DNA degradation and some cases of anemia^46,47^. Furthermore, recent works showed that chlorophyll derivatives, such as chlorophyllide, are also closely correlated to enhanced selectivity and improved cytotoxic activity against a range of carcinoma cells^48^.

### Total phenolic content

The ET extract presented the highest concentration (107.27 mg of gallic acid/100 g), followed by CA (96.82 mg of GAE/100 g) and HA (78.57 mg of GAE/100 g). Similar results (70.73 mg of GAE/100 g) were reported in fresh fruit extracts of wild-type *Bromelia anticantha* Berto^49^.

In general, phenolics have gained attention due to their antioxidant, antimutagenic, anticancer and anti-inflammatory capacities^50^. The aromatic benzene rings with substituted hydroxyl groups are responsible for their biological activity through the capacity to eliminate or absorb free radicals, and to chelate reactive oxygen species molecules formators. Furthermore, the effectiveness is generally proportional to the number of hydroxyl (OH) groups present in their aromatic rings^51^.

### Phenols content

The phenolic profile of aqueous extracts was evaluated in HPLC, in order to identify some antioxidants present in the solvents applied in the biological tests. The compounds were identified and quantified by comparing their retention times and absorption spectrum data in ultraviolet, which presented UV-band characteristic for gallic acid, p-coumaric acid, rutin, daidzein, quercetin, trans-cinnamic acid and genistein (Table 4).

**Table 4:** Retention times of phenolic compounds present in aqueous extracts of *N. procerum* Lindm.

	Hot Aqueous extract of *Nidularium procerum*				Cold Aqueous extract of *Nidularium procerum*		
Phenolic Compounds	Wavelength (nm)	Ret. Time	Area	Concentration (µg.g^−1^)	Ret. Time	Area	Concentration (µg.g^−1^)
Galic Acid *3,4,5-trihydroxybenzoic acid*	275	3.39	252476	2.35 ± 0.27	3.39	279619.3	2.63 ± 0.45
p-Coumaric Acid *3-(4-Hydroxyphenyl)-2-propenoic acid*	311	18.6	991292	4.39 ± 1.98	18.56	1117747.3	4.96 ± 0.22
Rutin *Quercetin-3-Rutoside*	357	21.7	129072	2.99 ± 0.33	21.72	143847	3.41 ± 0.23
Daidzein *Dihydroxyisoflavone*	260	23.4	161855	BDL*	23.1	170331	BDL
Quercetin 3’,4’,5’,7–*Tetrahydrixyflavon-3-ol*	370	25	30647	0.67 ± 0.09	25	33351.6	0.71 ± 0.04
Trans-Cinnamic Acid *Phenylacrylic acid*	275	25.3	322897	1.15 ± 0.09	24.87	272689.6	0.9 ± 0.2
Genistein *5,7,4 Trihydroxyisoflavone*	325	25.7	244231	18.75 ± 3.36	25.9	252668.3	19.44 ± 4.41

*BDL – Below detection limit.

The main component found was the isoflavone genistein (19.4 µg.g^−1^)—a compound belonging to the flavonoids class of phenols. Other flavonoids were also detected, such as tannin gallic acid (2.6 µg.g^−1^), flavone rutin (3.4 µg.g^−1^) and flavonol quercetin (0.7 µg.g^−1^). With the exception of genistein, that is usually found in leguminous^52^, flavonoids are characteristic of the Bromeliaceae family, having been reported in *Bromelia balansae* Mez, *E. spectabile* Mart. ex Schult. & Schult.f among others^53,54^.

Flavonoids and phenolic acids, such the p-coumaric and trans-cinnamic found in this study, are known by their antioxidant, antibacterial, antiviral, anti-inflammatory, cardio and hepatoprotective effects^55,56^. Genistein, in particular, have already being described for having chemotherapeutic potential against some tumor lines, such as prostate and gastric cancer^57,58^.

### Antioxidant activity of aqueous extracts

DPPH scavenging activity of hot aqueous (IC_50_: 0.18 ± 0.01 g.g^−1^ dry weight) and cold aqueous extract (IC_50_: 0.29 ± 0.01 g.g^−1^ dry weight) was dose-dependent, inhibiting up to 90% of free radicals and lower than the standard Trolox (IC 50: 18.30 ± 0.1 g.g^−1^ dry weight). However, HA extract also showed the highest value of Trolox equivalent (21.4 mg of Trolox.g^−1^ dry wheight), than CA extracts (18.83 mg of Trolox.g^−1^ dry weight) in 2,2-azinobis-3-ethyl-benzoatiazolin-6-sulfonic acid (ABTS) assay. According to total phenolic content of *N. procerum*, HA showed a significant difference to CA (p < 0,05), which is closely related to its higher antioxidant potential between the extracts.

In the human body, antioxidants are efficient against some metabolic disorders that compromise the corporal homeostasis, such as lipidic and protein peroxidation, DNA degradation and cell membrane alteration^59^. The antioxidant power of *N. procerum* extracts can be attributed to the molecular structure of compounds, inparticular polyphenolics. They can inhibit free radicals and chelate metals, acting in entire oxidative process. Gallic acid has three hydroxyl radicals attached in its aromatic ring, which are considered to be closely related to its antioxidant, cytotoxic and antiproliferative potential^60^. In comparison to other phenolic acids, trihydroxilated derivatives displays grater biological activities than phenolic acids with fewer hydroxyl radicals in their molecular structure, such as p-coumaric, trans-ferulic and trans-caffeic acids^61^. Furthermore, compounds such as chlorophylls, alkaloids and some fatty acids, appear to contribute to the antioxidant activity, due to their ability in delocalize the unpaired electrons of free radicals^62^.

### Immunomodulatory activity

**Figure 1:**
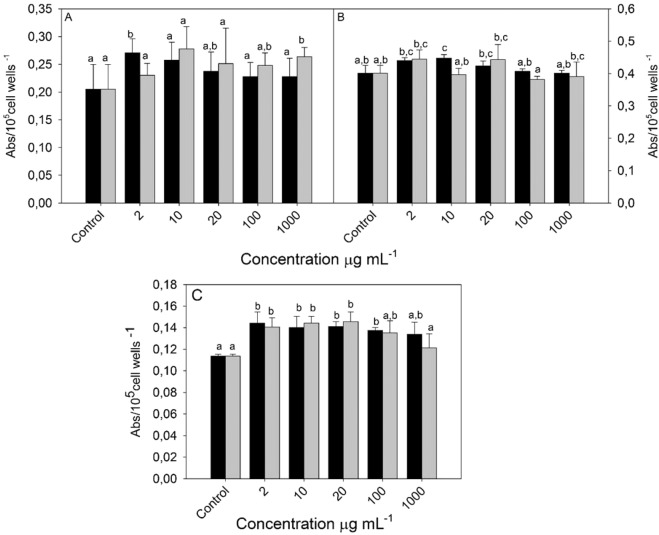
Macrophages adhesion (**A**), Phagocytosis activity (**B**) and Phagolysosomal formation (**C**) of macrophages (cell line RAW 264.7) treated with hot aqueous extract (dark bars) and cold aqueous extract (gray bars) of *Nidularium procerum* Lindm. Values are mean ± SE (n = 12). Different letters on bars indicate significant differences by Tukey test (p < 0,05).

Many plant extracts have long been described as possessing anti-inflammatory and immunomodulatory actions. The first line of human body defense against invading pathogens is the innate immune system, through macrophage cells. In the present study, murine macrophages were assessed *in vitro* by morphological indicators, such lysosomal volume, adhesion and phagocytic capacity, as well as metabolic activities of hydrogen peroxide and superoxide anion (Fig. 1). The macrophage adhesion response showed that the lowest concentration (2 µg.mL^−1^) of HA extract elicited a significant raise (p < 0.05) of about 32% of its activity (Fig. 1A**)**. On the other hand, CA extract showed no significant impact at 2 µg.mL^−1^, but increased the adhesion capacity at a higher concentration tested (1000 µg.mL^−1^) in over 28% of isolated macrophages. The adhesion alteration capacity of cells may be linked to the biological compounds present in HA extract. Fatty acids and polyphenols, especially flavonoids and tannins, can change properties of the plasma membrane, altering its fluidity capacity, as well as the distribution of adhesion molecules within the plasma membrane, such β1 (CD29) and β2 (CD18/11a, b, c) integrins^63^. β1 (CD29) and β2 (CD18/11a, b, c) are molecules involved in mediate the adherence of phagocyte cells to endothelium cell receptors.

Cells treated with 10 µg.mL^−1^ HA elicited a significant increase in the phagocytic capacity of macrophages (p < 0.05), while in CA extract, no significant effect was observed at all concentrations tested **(**Fig. 1B). Similar results were reported in cells treated with methanolic extracts of *Garcinia mangostana* L. and *Annona muricate* L^64^. Triterpenes, isocoumarins, steroids and flavones described in this study (*e.g*., stigmasterol, rutin, daidzein and genistein) can induce activities in phagocytic cells, by activating surface receptors, such Fc gamma (FcɣRI, FcɣRIIA and FcɣRIIB), starting the “zippering” phagocytosis^65^ and also increasing the expression of complement receptors-CR1, CR3 and CR4-promoting the “sinking” phagocytosis^66^. Furthermore, unsaturated fatty acids have previously shown to improve phagocytosis ability^67^, and according to Table 3, HA presented higher content of unsaturated fatty acids (73%) than CA (60%).

The phagolysosomal formation of macrophages was stimulated when the cells were treated with lower concentrations of extracts (p < 0,05), raising ~25% of neutral red uptake in both HA and CA, at 20 µg.mL^−1^ (Fig. 1C). This held the dose-dependent response and the proportional increase rate observed previously in phagocytosis activity. Lysosomes play the role of digesting intracellular components and also break down phagocytosed material, through the fusion of phagosome to hydrolase-containing lysosomal vesicles^68^, improving the defense cell mechanism.

Reactive oxygen species, such as hydrogen peroxide (H_2_O_2_) and superoxide anion (O_2_^−^), are generated in the first minutes of macrophage stimulation, during the so-called “respiratory burst”^69^. They are involved in the inflammation process, acting as efficient protectors; however, uncontrolled or excessive ROS production can further promote oxidative stress —a disruption in the redox balance system that contributes to damaging the body´s own cells and tissues^70^.

**Figure 2:**
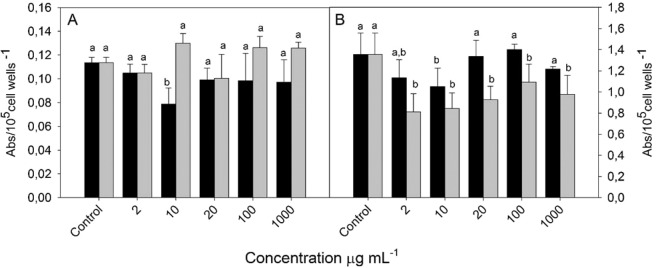
Hydrogen Peroxide Production (**A**) and Superoxide Anion Production (**B**) of macrophages (cell line RAW 267.4) treated with hot aqueous extract (dark ars) and cold aqueous extract (gray bars) of *Nidularium procerum* Lindm. Values are mean ± SE (n = 12). Different letters on bars indicate significant differences by Tukey test (p < 0,05).

HA extract (10 µg.mL^−1^) was capable to inhibit 36% the production of hydrogen peroxide (H_2_O_2_) in macrophage cells (Fig. 2A), while CA did not differ in comparison to the control at any concentration tested (p < 0.05). In the same way, HA (10 µg.mL^−1^) significantly reduced 38% of the production of superoxide anion (Fig. 2B), and different from that described in the H_2_O_2_ assay, CA was capable of inhibiting the production of O_2_^−^ in all concentrations (p < 0,05), with maximum reduction (40%) of activity at 2 µg.mL^−1^. This may be related to the potential of some flavonoids, such as rutin and quercetin, present in higher concentrations in CA extracts (3.41 and 0.71 µg.g^−1^ respectively) than HA, in the inhibition of xanthine oxidase and phosphoinositide 3-Kinase γ enzymes^71^. Furthermore, the higher concentration of H_2_O_2_ in relation to superoxide anion radical could be involved through the formation of enzyme-flavonoids hydrogen bonds, inhibiting the antioxidant activity of some peroxidase enzymes, such as catalase^72^.

Some molecules described in *N. procerum* extracts, especially flavonoids and derivatives, can act in different mechanisms of enzymes responsible for the oxidative burst in cells. The inhibition of ROS is related to their structure, the number and orientation of the hydroxyl group and the antioxidant potential of each compound; employing in its ability to permeate cell membrane and modulate the pathway signaling of NADPH-oxidase, phospholipase D, protein kinase C- (PKC) alpha, among others^73^. Plant extract compounds can also increase the expression of genes associated with the antioxidative system, such Cu/Zn-SOD, Mn-SOD, catalase, and GPx genes, suppressing oxidative stress by increasing antioxidant activity of enzymes^74^.

The ability to modulate all macrophage parameters found in *N. procerum* extract can be promising to help fight inflammation and even maintain cell homeostasis under different conditions. Leaf aqueous extract of *N. procerum* was also shown to interfere in different functions of host response capacity against injuries, such the inhibition of lipid body formation, PGE2 and cytokine production of *in vivo* pleural leukocytes^12^. Taken together, these data indicate that the substances described in the leaves of *N. procerum* proved to be efficient in modulating significant responses mediated by macrophages. This can be a potential alternative as a therapeutic agent applied in the prevention and treatment of pathologies related to the immune system. In addition, previous studies demonstrated that plant-derived compounds are able to alter the immunosuppressive status of patients, increasing antitumor immunity, promoting the proliferation of immune cells and accelerating macrophage phagocytosis^75^. To the best of our knowledge, there is no study on the anti-tumor activity of *N. procerum* extract. Studies were also carried out to evaluate the cytotoxic activity of *in vitro* cultured *N. procerum* Lindm against H295R cell line, a carcinoma with rare, heterogeneous malignancy and a very poor prognosis^76^.

### Antitumoral activity

**Figure 3:**
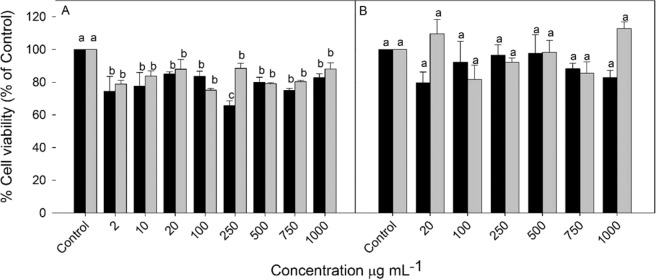
Viability percentage of tumor cells (H295R) (**A**) and non-tumor cells (VERO) (**B**) treated with different concentrations *of N. procerum* Lindm aqueous extracts within 24 h. Values are mean ± SE (n = 12). Different letters on bars indicate significant differences by Tukey test (p < 0.05). Black bars: Hot Aqueous Extract (HA). Grey bars: Cold Aqueous Extract (CA).

The key results obtained by MTT assay in H295R and the non-tumoral African green monkey kidney (VERO) cell lines exposed from 2 µg.mL^−1^ to 1000 µg.mL^−1^ for 24 h are summarized in Fig. 3. Both HA and CA showed significant decrease in tumor cell viability at all concentrations tested (Fig. 3A). The maximum mortality rate was 24,7% (CA at 100 µg.mL^−1^) and 34,4% -(HA at 250 µg.mL^−1^). On the other hand, there was no statistical difference among extracts and control in the viability of non-tumor cells (VERO) (Fig. 3B). The levels of extracts also showed no statistical differences, with no interaction among them. Molecules, such as phenolics described in this study, can either inhibit or stimulate the oxidative damage process, depending on the dose, structure, target molecule and environment^77^. In the present work, both HA and CA showed no cytotoxicity against normal cells, which makes these extracts promising as sources for the development of alternative drugs.

Antitumoral activity has already been found in some species or Bromeliaceae, such as *A. comosus* L.^78^, *Tillandsia recurvata* Baker^79^ and *Bromelia fastuosa* Lindl.^15^. The antitumoral activity was attibuted to cysteine proteinases (*e.g*., bromelain and fastuosain) as well as flavonoids, including penduletin, cirsimaritin and HLBT-100^33,79^. Biological compounds are related to the suppression of some metastatic markers, resulting in regulation of mitogen activated protein kinase and protein kinase B^80^. Genistein, present in higher amounts in both HA and CA (Table 4), is also related to the inhibition of protein tyrosine kinase and topoisomerase II, and elimination of oxygen free radicals^81,82^, inhibiting the bioavailability of sex hormones, platelet aggregation, angiogenesis, as well as modulating the apoptosis of malignant cell lines^83^. The biological activities of genistein are also related to the intramolecular hydrogen bonding formed by 5-hydroxyl and 4-ketonic oxygen^84,85^. These characteristics may be related to the cytotoxic potential of HA and CA extracts.

Furthermore, some tannins, alkaloids, saccharides and fatty acids, especially polyunsaturated, have proved to be efficient as antitumoral agents, inducing autophagy of cells and other pathways^86,87^. Until now, there have been only a few studies in the biological activity of *N. procerum* and none of them included the chemical compounds related to it, nor their potential as antitumor agents. Adrenocortical carcinoma is a rare and aggressive neoplasm with pour prognosis^88,89^, in which most patients diagnosed with advanced disease had a median survival time of less than 12 months and a 5-year survival rate of less than 15% among patients with metastatic disease^90^. In this scenario, the biological activity reported for *N. procerum* shows potential for the development of alternative treatments against adrenocortical carcinoma, which needs to be further explored through isolation and/or microencapsulation of bioactive compounds.

## Conclusions

The extracts obtained from the leaves of *in vitro* grown *N. procerum* were chemically characterized for the first time, showing the presence of phenolic compounds, steroids, fatty acids, polysaccharides, α-Tocopherol and scutellarein. These compounds showed good antioxidant activity and promoted the immunomodulation of murine macrophages. The crude extracts also showed potential against adrenocortical carcinoma cells, without cytotoxicity to non-tumoral cells, making it a potential candidate for alternative therapies against this tumoral line. However, further studies should be carried out to isolate and characterize *N*. procerum-derived compounds to improve cytotoxic activity as well as to prevent other human diseases caused by free radicals and other pathways.

## Methods

### Plant material and extraction

Plants were stablished and multiplicated *in vitro* according Lopes da Silva *et al*.^91^. Shoots (2 cm height) from clusters previously micropropagated *in vitro* were used as explants and subcultured *in vitro* to elongation and rooting for 90 days, free of plant growth regulators, up to the formation of a complete explant (basal and aerial part)-becoming available for extraction of leaves compounds, protocol adapted from Kim *et al*.^80^. Plantlets were removed from culture chambers and the leaves were cut into small pieces and 1 g of fresh leaf mass was macerated and extracted in 10 mL of specific solvent, over 24 hours, under 80 rpm agitation and 25 °C in a dark room. The extracts were filtered with Whatman n° 1 filter paper, lyophilized and stored at −20 °C for further characterizations and applications.

### Chemical characterization of *Nidularium procerum* extracts

**Figure 4:**
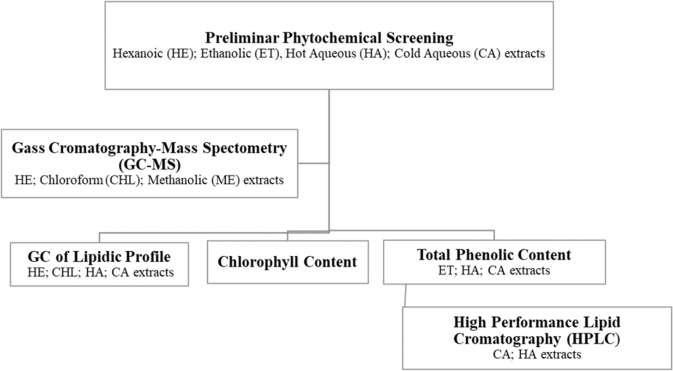
Flowchart of methodologies used in chemical characterzation of different extracts of *N. procum* Lindm.

The chemical investigation of *N. procerum* compounds were carried out in different solvents, in order to detect the maximum range of substances extracted as summarized in the flowchart below (Fig. 4**)**. The analyses were performed based on the results obtained in preliminary phytochemical screening and finally focused on aqueous extracts used in biological tests. The antioxidant, immunomodulatory and antitumoral potential of the aqueous fractions were explored due to their lack of toxicity and the low-cost of the process.

### Phytochemical screening

The phytochemical tests were carried out in four different extracts from *N. procerum*: hexane (Analytical standard-VETEC), ethanolic (Analytical standard-VETEC), hot aqueous (100 °C) and cold aqueous (25 °C). The screening was performed according to Iqbal *et al*.^92^. Identification of alkaloids was determined using Dragendorff, Mayer and Wagner´s test, reducing sugars using Fehling´s reagent, quinones by Bornträger´s test, saponins by permanent foam appearance, mucilage by gelatinous consistency after cooled, coumarins using Bajlet´s test, steroids/triterpenoids using Liebermann-Buchard´s test, resins by precipitation test, flavonoid by Shinoda´s test and tannins/phenols using Ferric Chloride´s test.

### Gas cromatography–mass spectrometry

The GCMS profiles of solvents with different polarity ranges (HE, CHL and ME) were obtained by electron impact with GC-MS-TQ Series 8040–2010 Plus (Shimadzu-Japan) equipped with a 95% PDMS and 5% Phenyl capillary column (model SH-Rtx-5MS; 30 m × 0.25 mm × 0.25 µm). The temperature program started at 50 °C, maintained for 2 minutes and raised at a flow of 7 °C.min^−1^ up to 280 °C, which remained constant for 15 minutes, for a total of 47 min of analysis. Helium was the carrier gas used, at a 1 mL.min^−1^, 88.3kPa column press and split ratio of 1:40. The solvent cut off was 2.5 min. The mass spectrometry range was 30–500 (m/z), at an ion source temperature of 250 °C. The chemical compounds were identified by comparison of the mass spectra present in NIST98/2014 and Wiley 7 data library.

### Lipid profile-gas chromatography

Fatty acid profile of CHL, HE, HA and CA extracts were analyzed using a Shimadzu chromatograph (GC 2010 *Plus*), a capillary column (SH-Rtx-Wax - Shimadzu: 30 m × 0.32 mm × 0.25 μm), flame ionization detector (FID) and split injection mode (1:10). The injector and detector temperatures were 240 °C and 250 °C, respectively. The oven temperature was programmed to start at 100 °C during 5 min, followed by an increase up to 240 °C at a rate of 4 °C.min^−1^ and maintained at this temperature for 5 min. The carrier gas was Helium at 32.5 cm^3^.min^−1^. The samples were prepared according to the official method (Ce 2–66) of the American Oil Chemist’s Society (AOCS, 1998) to convert triacylglycerol and free fatty acid of samples into fatty acid methyl esters (FAMEs). FAMEs were identified by comparison with retention times of the standard mixture FAMEs (Supelco, MIX FAME 37, St. Louis, MO 63103, USA). The quantification of fatty acids was conducted by area normalization procedure. Results were expressed as percentage of each individual fatty acid present in the sample.

### Chlorophyll quantification

Fresh leaves (5 g) were macerated in 10 mL acetone (P.A. VETEC). The solution was filtered with Whatman n° 1 filter paper and stored at −6 °C for five minutes. The absorbance was measured with spectrophotometry at 470, 662, 645, and 652 nm to chlorophyll a, b, relation between and chlorophyll a/b, respectively^93^. The assays were carried out in triplicate and the results were expressed in μg.g^−1^ of fresh weight.

### Total phenol content

Total phenolic contents of ET, HA and CA extracts were determined using Folin-Ciocalteu method^94^ and the standard curve was performed using 0.39, 3.9, 7.8, 15.6, 31.2, 62.5 and 125 μg.mL^−1^ of gallic acid. The results were expressed in mg of gallic acid equivalent (GAE) in 100 g of fresh weight.

### Phenolic content - high performance liquid chromatography

The phenolic content of aqueous extracts was separated in HPLC using an Agilent Technology 1200 Series system, coupled to a diode array detector (DAD) at wavelengths 235, 260, 275, 280, 290, 311, 357, 370 nm and a scanning from 190 nm to 600 nm. A ZorbaxElipse XDB-C18 (4,6 ×150 mm, 5-micron) column was used at 0,7 mL min^−1^ flow. The mobile phase was 2,5% acetic acid (solvent A) and methanol (solvent B). The elution gradient was carried out as follows: 90% A/10% B, 0–13 min; 75% A/25% B, 13–28 min; 15% A/85% B, 28–32 min; 10% A/90% B, 32–36 min. Chlorogenic acid, caffeic acid, ferulic acid, tocopherol, genistein, transcinnamic acid, catechin, rutin, p-coumaric acid, gallic acid, resveratrol and epicatechin (SIGMA) were used as standards. To obtain the calibration curve, all standard reagents were solved in mobile phase and used at 1, 2, 5, 8 and 10 ppm. The samples were microfiltered trough a hydrophilic membrane GV (Durapore) made of polyvinylidene difluoride (PVDF), with a pore size of 0,22 μm. The resulting chromatogram values were plotted and a linear equation was generated by calculating the average of triplicate runs for each compound. The equations were used to quantify the phenolic compound contents of the samples. The injection volume was 10 µL. All the assays were also performed in triplicate.

## *In vitro* antioxidant activity

### Scavenging ability on DPPH

The antioxidant potential of the aqueous extracts was determined by their ability of quenching the free radical DPPH^69^. A Trolox (Sigma) standard solution was diluted from 0.25 to 25 mg/ml and used as positive control to the assay, mixing 200 μL of each concentration in 800 μl of 0.004% methanol solution of DPPH. After 30 min of incubation in absence of light at room temperature, the absorbances were read against blank at 517 nm using a SP-2000 spectrophotometer. The same protocol was used for the HA and CA treatments. DPPH solution was used as negative control with the solvent extraction. Tests were carried out in triplicate and the percentage of free radical inhibition was calculated by the following Eq. (1):

1 $$ \% {\rm{I}}=({{\rm{A}}}_{{\rm{blank}}}\mbox{--}{{\rm{A}}}_{{\rm{sample}}}/{{\rm{A}}}_{{\rm{blank}}})\times 100$$

Where A_blank_ is the negative control and A_sample_ is the absorbance of extracts. The results were expressed in extract concentration producing 50% inhibition (IC 50%), calculated from the graph of the DPPH scavenging effect against the extract concentration.

### ABTS assay

The ABTS assay was carried out using a radical cation decolorization protocol^95^. The ABTS radical had to be pre-formed by the reaction between 5 mL ABTS 7 mM (Sigma) with 88 μL of 140 mM potassium persulfate, stored in the dark at room temperature for 16 hours. The ABTS solution (1 mL) was previously diluted in 50 mL of ethanol P.A. (Alphatec) to obtain an absorbance of 0.700 at 734 nm. In absence of light, 10 μL of each aqueous plant extract were added to 500 μL ABTS solution. After 6 minutes, the absorbance was read in the spectrophotometer (SP 2000) at 734 nm. Distilled water was used as blank and as negative control. All measurements were carried out in triplicates. The scavenging capability of tests compounds was calculated using the following Eq. (2):

2 $${\rm{ABTS}}\,{\rm{Scavenging}}\,{\rm{activity}}\,( \% )=(1-({{\rm{\lambda }}}_{734-{\rm{Sample}}}/{{\rm{\lambda }}}_{734-{\rm{Control}}}))\times 100$$

Where λ_734-Sample_ is the absorbance of control without radical scavenger and λ_734-Control_ the remaining ABTS in the presence of scavenger. Trolox was used as standard.

### Immunomodulatory activity

Macrophage activity was assessed by its reactive oxygen species production - superoxide anion and hydrogen peroxide, cell adhesion, phagocytic efficiency and phagolysosomal formation^96^. Murine macrophages cells were cultured in Dulbecco’s Modified Eagle’s medium (DMEM-Sigma Aldrich) supplemented with 10% fetal bovine serum (FBS - Gibco and 1% antibiotic solution (10.000 U.mL^−1^ penicilin and 10 mg.mL^−1^ streptomycin - Gibco^®^), maintained in a humidified atmosphere with 5% CO_2_ at 37 °C until 80–90% confluence was reached. The cells were divided at 10^5^ cells/well in 96-well plate (Biofil) and exposed into the following experimental groups: cells without treatment (C), Hot Aqueous (HA) and Cold Aqueous Extract (CA), both at concentrations 2, 10, 20, 100 and 1000 μg.mL^−1^ for 24 h at same conditions of growing. The analyses were performed in 12 repetitions.

### Antitumoral activity

H295R and VERO cells were cultured in DMEM F-12 (Sigma Aldrich) medium supplemented with 10% fetal bovine serum (Gibco) and 1% antibiotic solution (Gibco). They were incubated in a CO_2_ incubator at 37 °C, with humidified air (95%) and CO_2_ (5%) until 80–90% confluence was reached. The cells were divided in 10^5^ cells/wells in a 96-well plate (Biofil) and exposed into the following experimental groups: cells without treatment (C), Hot Aqueous and Cold Aqueous Extract, both at concentrations 2, 10, 20, 100 250, 500, 750 and 1000 μg.mL^−1^ for 24 h at the same growing conditions. The analyses were performed in 12 repetitions^97^.

### Statistical analysis

The data were expressed as mean ± standard error and analyzed by One Way Anova followed by Tukey´s test using Graph Pad Prism 6 software. Differences between means at the 5% of confidence interval (p < 0.05) were considered significant.
